# Catatonia in Juvenile Systemic Lupus Erythematosus: A Case Series

**DOI:** 10.31138/mjr.311223.cij

**Published:** 2024-07-24

**Authors:** Mahabaleshwar Mamadapur, Sabarinath Mahadevan, Ponniah Subramanian ArulRajamurugan

**Affiliations:** 1 Dept. of Clinical Immunology and Rheumatology, JSS Medical College and Hospital, JSS Academy of Higher Research, Mysore, Karnataka, India; 2 O.M. Health Care, Tamil Nadu, India; 3 Madras Medical College, Chennai, Tamil Nadu, India

**Keywords:** juvenile SLE, movement disorder, neuropsychiatric lupus

## Abstract

Neuropsychiatric systemic lupus erythematosus (NPSLE) presents a significant diagnostic and therapeutic challenge due to its varied clinical manifestations. The prevalence of NPSLE ranges widely, reported between 37% and 95% in different case series, reflecting this condition’s complex and heterogeneous nature. Here we report three cases of juvenile systemic lupus erythematosus (SLE) presenting with catatonia as a rare neuropsychiatric manifestation.

Case 1 is a 15-year-old male with fever, and pancytopenia, diagnosed with SLE and subsequent development of catatonia. Case 2 is a 14-year-old female with a history of SLE presenting with altered sensorium, restlessness, and catatonia. Case 3 is a 15-year-old male with SLE exhibiting abnormal behaviour and catatonia.

Treatment strategies for these cases include high-dose steroids, immunosuppression, and benzodiazepines. This case series emphasises the importance of a multidisciplinary approach, prompt diagnosis, aggressive treatment, and vigilant follow-up to optimise outcomes in these vulnerable paediatric patients.

In conclusion, this case series contributes to the literature on catatonia in paediatric SLE, emphasising the need for expanded awareness, early recognition, and comprehensive management strategies. Further research is warranted to refine predictive factors and establish optimal maintenance protocols for this complex neuropsychiatric manifestation.

## INTRODUCTION

Neuropsychiatric systemic lupus erythematosus (NPSLE) presents a significant diagnostic and therapeutic challenge due to its varied clinical manifestations. The prevalence of NPSLE ranges widely, reportedly between 37% and 95% in different case series, reflecting this condition’s complex and heterogeneous nature.^1^ In 1999, the American College of Rheumatology (ACR) established case definitions for diagnosing 19 distinct NPSLE syndromes, providing a framework for clinicians to identify and manage these manifestations.^2^

While the ACR case definitions have been valuable, they do not encompass all possible neuropsychiatric manifestations of lupus. Notably, catatonia, a behavioural syndrome characterised by abnormal movements and immobility, was not explicitly included in the ACR definitions.^3^ Despite this omission, there have been documented cases of catatonia in systemic lupus erythematosus (SLE), highlighting the need for a broader consideration of neuropsychiatric symptoms in lupus patients.^4^ Catatonia is a complex syndrome defined by the Diagnostic and Statistical Manual of Mental Disorders, Fifth Edition (DSM-5), which outlines specific criteria for its diagnosis.^5^ The DSM-5 requires the presence of three or more out of a list of characteristic features, including agitation, grimacing, mannerisms, posturing, stupor, stereotypy, negativism, mutism, waxy flexibility, cataplexy, echolalia, and echopraxia.^6^

In this report, we present three cases of juvenile SLE in which catatonia was a prominent neuropsychiatric manifestation. These cases underscore the importance of recognising and understanding catatonia as a potential SLE complication, even without explicit inclusion in established diagnostic criteria. Expanding our awareness of the diverse neuropsychiatric presentations of lupus can improve early detection and management of these challenging cases.

## CASE PRESENTATIONS

### Case 1

A 15-year-old male was admitted with a 7-day history of fever, bleeding from gums, and melena. Initial investigations revealed pancytopenia, prompting an investigation for malignancy. Bone marrow aspiration and biopsy, however, yielded normal results. Subsequently, the patient was referred to the Rheumatology department due to persisting symptoms.

Rheumatological workup demonstrated positive findings with a speckled 3+ antinuclear antibody (ANA) on Hep2 assay and an elevated anti-double-stranded DNA (dsDNA) level of 364.4 IU/ml. The antiphospholipid antibody (APLA) workup was normal. The patient was administered pulse Methyl Prednisolone and planned to start immunosuppression.

During hospitalisation, he developed altered sensorium, char-acterised by decreased speech and interaction with family members, an inability to recognise relatives, decreased oral intake, grimacing, mutism, waxy flexibility, and posturing **(Video 1)**. He also had an episode of generalised tonic-clonic seizures. MRI Brain and cerebrospinal fluid (CSF) analysis were normal, and culture was negative. Blood culture and urine analysis were negative for microorganisms. Viral markers were negative. Screening for viral markers (HIV, HBsAg, HCV) were negative. Viral encephalitis panel, CSF Multiplex PCR, CSF for Indian INK preparation and fungal culture, and VDRL tests were negative. Chest X-ray was normal. C3 and C4 levels were low. The lab parameters are summarised in **Table 1**. Psychiatry and neurology consultation was sought. The patient satisfied the DSM-5 criteria for catatonia. His Systemic Lupus Erythematosus Disease Activity Index 2000 (SLEDAI-2K) score was 30.

**Table 1.** Summary of clinical and laboratory characteristics of the cases.

	**Case 1**	**Case 2**	**Case 3**
**Gender**	Male	Female	Male
**Age at presentation**	14	15	14
**Disease duration (yrs)**	1	2	1
**Catatonic features**	grimacing, mutism, waxy flexibility, and posturing	mannerisms, posturing, stupor, stereotypy, negativism, mutism, waxy flexibility, cataplexy, echolalia, and echopraxia	posturing, stupor, stereotypy, negativism, mutism
**Haemoglobin (g%)**	9.1	8.4	11
**Total leucocyte count (per cumm)**	2570	4190	5900
**Differential count (per cumm)**	N86.6, L11.5	N 72, L12	N78, L12
**Platelet count (per cumm)**	59000	90000	1.43L
**BUN (mg/dl)**	35	25	81
**Creatinine (mg/dl)**	0.5	0.55	0.6
**Na+/K+**	139/3.8	130/3.5	132/5
**SGOT/SGPT (mg/dl)**	38/20 576/149 (2^nd^ visit )	28/32	34/13
**Urine Routine**	Albumin 1+	Normal	Normal
**24 hr Urine Protein (mg)**	0.5	0.3	0.3
**ANA (IIF)**	Speckled 3+	Homogenous 2+	Speckled 3+
**ANA profile**	Negative	Ribosomal P	-
**Anti ds DNA**	364.4 (1^st^) 529 (2^nd^)	104	-
**C3 (mg/dl)**	43	92	18.17
**C4 (mg/dl)**	4	36	3.92
**Antiphospholipid antibody**	Acl IgG/IgM:30/36 Β2GPI IgG/IgM:2.92/4.72	Acl IgG/IgM:24.4/25.3 Β2GPI IgG/IgM:7/27	-
**Lupus Anticoagulant**	Negative	Negative	-
**CSF analysis**	Normal	Normal	Normal
**MRI Brain**	Chronic haemorrhages in the posterior parietal region	Normal	Normal
**Management**	Steroids, Cyclophosphamide, lorazepam	Steroids, Cyclophosphamide, lorazepam	Steroids, Cyclophosphamide, lorazepam

In response to the catatonic features and high disease activity, the patient was started on Cyclophosphamide, as per the National Institutes of Health (NIH) protocol. Lorazepam was administered at 6mg/day in divided doses, alongside antiepileptic therapy with Phenytoin (20mg/kg), to manage the catatonia. His symptoms improved and he was discharged after a two-week hospital stay. The patient defaulted follow-up for 5 months and presented with fever, jaundice (total/direct bilirubin:13.9/12.5 mg/dl, transaminases: 576/149), seizures, and recurrent catatonic features including stupor, posturing, and grimacing. Anti-dsDNA titre was 529 IU/ml. Viral screening and autoimmune hepatitis workup were normal. **(Table 1)**. The patient was administered 75% of the usual dose of Cyclophosphamide, resulting in improvement of symptoms and liver function tests. He is currently under regular follow-up, continuing Cyclophosphamide injections as part of his management plan **(Video 2)**. (**Video 3**: Follow-up)

### Case 2

A 14-year-old female with systemic lupus erythematosus (SLE) presented with a one-week history of altered sensorium, restlessness, mutism, excessive crying, fearfulness, waxy flexibility, and posturing. Two years prior, she had experienced hair loss, oral ulcers, breathlessness, and constitutional symptoms. Her 2D echocardiogram showed global left ventricular hypokinesia with an ejection fraction (EF) of 40%. She was commenced on intravenous Cyclophosphamide as per NIH protocol. But she was lost to follow-up.

During present admission, further investigations, including a lumbar puncture (LP), cerebrospinal fluid (CSF) analysis, blood and urine cultures, and CT brain imaging yielded normal results. Laboratory tests showed an anti-double-stranded DNA (anti-dsDNA) level of 104.3 IU/ml, with normal complement levels (C3 and C4).

Her present SLEDAI-2K score was 20. She was diagnosed with SLE with catatonia and was treated with intravenous Methylprednisolone, followed by Cyclophosphamide as per NIH protocol along with lorazepam at a dose of 6mg per day. The patient showed improvement in her symptoms and was discharged with plans for regular follow-up. She did not experience a relapse of symptoms during subsequent visits.

### Case 3

A 15-year-old male, diagnosed with systemic lupus erythematosus (SLE), was brought to the hospital with a two-day history of fever and altered sensorium. His parents reported a sudden onset of abnormal behaviour, reduced oral intake, occasional disorientation, and non-compliance with commands. He had 1–2 episodes of involuntary micturition, a significant decrease in sleep, and reduced speech output. His parents observed that he maintained uncomfortable positions for extended periods without apparent reason. There were no reported seizures. The patient had a history of being on Azathioprine and hydroxychloroquine (HCQ) but had recently discontinued his medication. The patient displayed mutism, posturing, and rigidity, leading to the diagnosis of neuropsychiatric SLE (NPSLE) with catatonia. His SLEDAI-2K score was 16. Lumbar puncture (LP) and cerebrospinal fluid (CSF) analysis, as well as magnetic resonance imaging (MRI) of the brain, yielded normal results. Urine and blood cultures and viral encephalitis panel were negative. The patient was treated with intravenous Methylprednisolone and Cyclophosphamide as per (NIH) protocol. Following the treatment plan, the patient’s symptoms improved, and he was subsequently discharged and is on regular follow-up.

## DISCUSSION

Catatonia is a behavioural syndrome that was historically associated with schizophrenia, although it can occur in various psychiatric and non-psychiatric disorders. Nonpsychiatric causes can be due to infections (CNS or systemic), delirium, stroke, nonconvulsive status epilepticus, Neuroleptic malignant syndrome, and Anti-N-methyl-D-aspartate (NMDA) receptor encephalitis.^5^

Neuropsychiatric manifestations in systemic lupus erythematosus (SLE) are challenging to manage and are associated with significant morbidity and mortality. Catatonia is an uncommon but severe neuropsychiatric manifestation of SLE that requires prompt recognition and treatment. During the natural course of SLE, catatonia can present as an initial manifestation or as a lupus relapse.

The three paediatric cases presented demonstrated typical features of catatonia in SLE, including mutism, posturing, grimacing, and waxy flexibility. All 3 cases satisfied the DSM V criteria for diagnosis of catatonia. Investigations were undertaken to rule out other aetiologies. Disease activity was assessed using the SLEDAI-2K score. All three patients showed improvement with high-dose corticosteroids, immunosuppressants like Cyclophosphamide, and supportive therapy with benzodiazepines.

These findings align with existing literature on paediatric catatonic lupus. Fragoso-Loyo et al. described 3 cases of paediatric catatonic lupus that similarly improved with corticosteroids, cyclophosphamide, and lorazepam.7 Grover et al. reported a 22-year-old female with lupus who presented with catatonic symptoms at the time of relapse of SLE. She was managed with lorazepam, steroids, and cyclophosphamide.^8^

Annabel Boeke et al. reported two cases of SLE with catatonia and reviewed 35 additional cases from the literature. In these cases, immunosuppressive therapy was commonly administered for the underlying SLE. Symptomatic catatonia treatment included benzodiazepines (81% of patients) and electroconvulsive therapy (38%). Catatonia should be considered in NPSLE, managed by addressing the autoimmune condition, avoiding exacerbating factors, and providing symptomatic relief with benzodiazepines, with electroconvulsive therapy as an option for refractory cases. This study contributes to clinical awareness and optimal management of catatonia in NPSLE.^9^ In another case series, Sundaram et al. presented two cases of SLE with catatonia, both showing improvement with immunosuppressive treatment. The first case had fever, pancytopenia, toxic epidermal necrolysis (TEN)-like rash, and later developed catatonia and macrophage activation syndrome (MAS). The second case presented with acute cutaneous lupus erythematosus (ACLE), fever, alopecia, polyarthralgia, ne phritis, cytopenias, and catatonia.^10^ They reviewed the articles and noted that 37 cases have been described so far. The age of presentation varied from 14–46 years. Catatonia usually developed one month to thirteen years after the start of SLE, but it can also be the first symptom in certain cases. Two cases manifested with psychiatric manifestations initially. Patients who developed catatonia did not consistently exhibit specific clinical or lab features, although nephritis was infrequent, occurring only in three cases. Six cases were positive for anti-ribosomal antibodies. Timely recognition and treatment with immunosuppression, alongside benzodiazepines with or without electroconvulsive therapy (ECT), proved essential for the successful management of these cases.^10^

Case 1 highlighted relapse after 5 months despite initial improvement in our study. This tendency for relapse was described by Brelinski et al., who observed recurrence rates of up to 50% in paediatric catatonic lupus patients. Hence, diligent follow-up is critical.^11^

## CONCLUSION

In conclusion, these cases align with existing evidence on paediatric catatonic lupus, a rare but serious neuropsychiatric manifestation of SLE. Prompt diagnosis, aggressive immunosuppression, supportive therapy, and careful long-term monitoring are key to optimising outcomes in these vulnerable patients. Integrating a multidisciplinary approach and maintaining adherence are also pivotal, given the risk of recurrence. Further research could elucidate predictive factors and optimal maintenance regimens.
